# Insights into early pathogenesis of sporadic Alzheimer’s disease: role of oxidative stress and loss of synaptic proteins

**DOI:** 10.3389/fnins.2023.1273626

**Published:** 2024-01-08

**Authors:** Mubeen A. Ansari, Muddanna Sakkattu Rao, Aishah Al-Jarallah

**Affiliations:** ^1^Department of Pharmacology and Toxicology, College of Medicine, Kuwait University, Jabriya, Kuwait; ^2^Department of Anatomy, College of Medicine, Kuwait University, Jabriya, Kuwait; ^3^Department of Biochemistry, College of Medicine, Kuwait University, Jabriya, Kuwait

**Keywords:** streptozotocin, hyperglycemia, oxidative stress, synaptic proteins, cognitive behavior, Alzheimer’s disease

## Abstract

Oxidative stress, induced by impaired insulin signaling in the brain contributes to cognitive loss in sporadic Alzheimer’s disease (sAD). This study evaluated early hippocampal oxidative stress, pre- and post-synaptic proteins in intraperitoneal (IP) and intracerebroventricular (ICV) streptozotocin (STZ) models of impaired insulin signaling. Adult male Wistar rats were injected with STZ, IP, or ICV, and sacrificed 1-, 3-, or 6-weeks post injection. Rat’s cognitive behavior was assessed using Morris water maze (MWM) tests at weeks 3 and 6. Hippocampal synaptosomal fractions were examined for oxidative stress markers and presynaptic [synapsin I, synaptophysin, growth-associated protein-43 (GAP-43), synaptosomal-associated protein-25 (SNAP-25)] and postsynaptic [drebrin, synapse-associated protein-97 (SAP-97), postsynaptic density protein-95 (PSD-95)] proteins. IP-STZ and ICV-STZ treatment impaired rat’s cognition, decreased the levels of reduced glutathione (GSH) and increased the levels of thiobarbituric acid reactive species (TBARS) in a time dependent manner. In addition, it reduced the expression of pre- and post-synaptic proteins in the hippocampus. The decline in cognition is significantly correlated with the reduction in synaptic proteins in the hippocampus. In conclusion, impaired insulin signaling in the brain is deleterious in causing early synaptosomal oxidative damage and synaptic loss that exacerbates with time and correlates with cognitive impairments. Our data implicates oxidative stress and synaptic protein loss as an early feature of sAD and provides insights into early biochemical and behavioral changes during disease progression.

## 1 Introduction

Alzheimer’s disease (AD) is characterized by multiple cognitive deficits including loss of learning/memory that results from neurodegeneration in the cerebral cortex (Santos et al., 2012; Yassine et al., 2013; Cheng et al., 2014). Role of oxidative stress and loss of synaptic proteins in cerebral cortical tissues has been shown to precipitate in behavioral anomalies (Ansari and Scheff, 2010, 2011; Scheff et al., 2015, 2016). Beside the genetic factors in familial AD (fAD) several risk factors were implicated in the development of AD in early age, termed as sporadic AD (sAD), the most prevalent form of the disease (Jayaraj et al., 2020; Latina et al., 2021). AD is characterized by the accumulation of amyloid-β (Aβ) plaques and hyperphosphorylated tau (p-tau) protein in the brain, leading to synaptic dysfunction and cognitive impairments.

Recent research identified a close association between AD and diabetes whereby impaired insulin signaling in the brain results in similar pathophysiological characteristics of sAD. This includes impaired glucose/energy-homeostasis, enhanced oxidative stress and inflammation and cognitive deficits (Rajasekar et al., 2017; Gocmez et al., 2019; Gao et al., 2020; Huang et al., 2020; Ide et al., 2020; Latina et al., 2021). We have recently reported increased oxidative stress over time in hippocampal synaptosomes following IP and ICV STZ administration (Ansari et al., 2023). Prolonged exposure to elevated levels of oxidative stress and inflammation in the brain exacerbates insulin resistance and leads to progressive tissue damage (Rajasekar et al., 2017; Jayaraj et al., 2020; Latina et al., 2021). Moreover, STZ induced insulin resistance and/or impaired insulin signaling in the brain results in increased free radical production and reduced energy metabolism (Gasparini et al., 2002). In parallel, activation of microglial/astroglia (inflammation) exacerbates the generation of free radicals. Oxidative stress and inflammation together result in synaptic loss and disrupt hippocampal neuronal circuits leading to cognitive impairments (Vajda, 2002; Shoham et al., 2003; Ishrat et al., 2006, 2009; Ansari et al., 2023). In addition, hyperglycemia affects different neuronal pathway/systems including cholinergic system (Mundinger et al., 2015), dopaminergic system (Rebolledo-Solleiro et al., 2016) and gamma aminobutyric acid (GABA)-ergic system (Martin et al., 1988). Alterations in different neuronal pathways result in reduced brain functions along with loss of hippocampal synaptic plasticity, expressed as deficits in long-term potentiation (LTP) (Momeni et al., 2021) and enhanced long-term depression (LTD) (Gardoni et al., 2002). Synaptic plasticity and functions are mediated by synaptic proteins, ionotropic and metabotropic receptors, including α-amino-3-hydroxy-5-methyl-4-isoxazolepropionic acid receptors (AMPAR) and glutamate receptors (GluR). Disease progression results in the decreased expression of presynaptic and postsynaptic proteins, like synapsin and postsynaptic density protein 95 (PSD-95), in the hippocampal tissue (Gardoni et al., 2002; Wang and Zhao, 2016). In chronic cases this is aggravated by increased lipid peroxidation (Mooradian et al., 1990) and reduced AMPA binding to glutamate receptor-1 (GluR1), prominently in the hippocampus (Gagne et al., 1997). Moreover, reduced hippocampal LTP (Franzon et al., 2005) results in the progressive loss of cognitive behavior in diabetic animals (Wang et al., 2015). Similar, yet greater effects are observed upon direct intracerebroventricular (ICV) administration of STZ (Shonesy et al., 2012; Chen et al., 2014; Cheng et al., 2019). In this model, cognitive loss is observed 2 weeks post ICV-STZ injection (Ansari et al., 2023). In addition, augmented oxidative stress, inflammation, apoptotic cell death, mitochondrial dysfunction, and reduced expression of synaptic proteins are detected in the hippocampus and cerebral cortex (Rai et al., 2014). These anomalies are aggravated over the time via the downregulation of insulin receptors (IRs) signaling in the hippocampus and cerebral cortex (Wang et al., 2018) leading to decreased hippocampal LTP (Hashemi-Firouzi et al., 2017), neuronal dysfunction (Shonesy et al., 2012; Gao et al., 2014; Lu et al., 2017), and loss of synaptic proteins (Baltaci et al., 2022).

As reviewed above, even though several studies have attempted to explore the mechanism of impaired insulin signaling as primary players in the development of sAD, there is no convincing mechanism yet established. In the current study, we examined early changes in the levels of oxidative stress and the expression of major presynaptic [synapsin-I, synaptophysin, growth-associated protein-43 (GAP-43), synaptosomal-associated protein-25 (SNAP-25)] and postsynaptic [drebrin, synapse-associated protein-97 (SAP-97), and postsynaptic density protein-95 (PSD-95)] proteins in hippocampal synaptosomes from IP-STZ and ICV-STZ treated rats (two different models of imparted insulin signaling). Our data indicate declined cognition, enhanced oxidative stress and reduced expression of pre- and post-synaptic proteins in response to STZ administration early in the course of the disease (3–6 weeks). STZ administration via the ICV route demonstrated profound deterioration in the parameters tested compared to the IP route of STZ administration. Finally, reduced expression of synaptic proteins is correlated with loss of cognition in STZ treated rats. Our study provides mechanistic insights into early changes in hippocampal synaptosomes in response to impaired insulin signaling as primary players in the development of sAD.

## 2 Materials and methods

### 2.1 Chemicals

Pierce^®^ BCA protein assay reagents and protease inhibitors (mini tablets) were purchased from Thermo Fisher Scientific, Inc. (Pittsburgh PA, USA). Electrophoresis apparatus and applied chemicals were purchased from Bio-Rad Laboratories, Inc. (Hercules CA, USA). Mouse monoclonal anti synapsin-I (sc-376623), synaptophysin (sc-17750), GAP-43 (sc-17790), SNAP-25 (sc-376713), drebrin (sc-374269), SAP-97 (sc-9961), PSD-95 (sc-32290), and β-actin (sc-47778) antibodies were purchased from Santa Cruz Biotechnology (Santa Cruz, CA, USA). Alkaline phosphatase conjugated anti-mouse (A3562) secondary antibody and all other chemicals/reagents, including streptozotocin (STZ), were purchased from Sigma (St. Louis, MO, USA) unless stated otherwise.

### 2.2 Animals

Three months old male Wistar rats (365–400 g), in house bred from the same batch of breading, were used in this study. Rats were housed at the Animal Resources Center (ARC), Collage of Medicine, Kuwait University and had free access to food and water. The rats were housed under controlled conditions of temperature 25 ± 2°C, humidity 50%, and 12-h of light/dark cycle. The use of animals and the surgical procedure were approved by Health Sciences Center Animal Research Ethics Committee (HSC-AREC), Kuwait University. HSC-AREC follows the recommendations of NIH guidelines for the care and use of laboratory animals.

### 2.3 Animal groups and experimental design

We followed a similar experimental design as in our recent publication (Ansari et al., 2023). Briefly, two rat models of impaired insulin signaling in the brain were used to assess cognitive performance and changes in pre- and post-synaptic proteins in hippocampal synaptosomes, namely intraperitoneal streptozotocin (IP-STZ) and intracerebroventricular streptozotocin (ICV-STZ) rat models. The groups were randomized, assessed by investigators who were not aware with different groups of the treatment. One hundred twenty rats were divided into four sets (30 rats in each set) that either received IP-Vehicle, IP-STZ, ICV-Vehicle, or ICV-STZ. Each set was further divided into three groups (*n* = 10/group) that were euthanized at three different time points [1 week (1W), 3 weeks (3W), or 6 weeks (6W)] after the administration of the vehicle or STZ. Rats in the IP-Vehicle groups received a single IP injection of 0.5 ml of the vehicle (citrate buffer, 0.1 M, pH 4.6) and rats in IP-STZ groups received a single injection of STZ (55 mg/kg) on the first day of experiment. Blood glucose levels in IP-STZ treated rats were checked on the 4th day and monitored every 7th day. A minimum of 17.5 mmol/l glucose was used as a cutoff for hyperglycemia/diabetes. On day one, the ICV-Vehicle rats received a single injection of 5–7 μl vehicle (sterile citrate buffer, 0.1 M, pH 4.6) prepared in artificial cerebral spinal fluid (CSF), while ICV-STZ rats received a single injection of 5–7 μl STZ (1.0 mg/6 μl), according to their body weight, into each of the left and right lateral ventricles. This dose of STZ (3.0 mg/kg body weight) was selected based on previous publications (Ishrat et al., 2006, 2009; Ansari et al., 2023). ICV infusion of the vehicle or STZ into the lateral ventricles was performed gradually over a period of 20 min to allow proper diffusion into the CSF in cerebral ventricles. In ICV-STZ injected rats, blood glucose did not change on day 4 and after, was monitored on every 7th day. At 3W and 6W post administration of the vehicle or STZ (IP and ICV) rats were assessed for cognitive performance using Morris water maze (MWM) for spatial learning and memory. Rats in different groups were sacrificed 1W, 3W and 6W after commencement of the experiment. Brains were removed quickly, hippocampi (left and right) were dissected, snap frozen in liquid nitrogen and stored at −80°C until processing for biochemical analysis.

### 2.4 Surgical procedure for ICV- STZ/vehicle injection

Vehicle or STZ was injected into the lateral ventricles as described previously (Ishrat et al., 2006, 2009; Ansari et al., 2023). Surgical procedure was performed in aseptic conditions. All tools and materials applied in the surgery were autoclaved and workstation and stereotaxic apparatus were sterilized with 70% ethanol. In between surgical procedures small hand tools were sterilized using glass-bead sterilizer (GERMINATOR™ 500). Each rat was anesthetized with an intramuscular injection of ketamine/xylazine hydrochloride cocktail (ketamine-60 mg/kg, xylazine-5 mg/kg). The anesthetized rat was placed on a stereotaxic apparatus (fixed with the help of ear bars and incisor bar) and a 2 cm long skin incision was made to expose the skullcap. Two bur holes, one on either side of the midline (1.5 mm from midline, 0.8 mm posterior to bregma) were drilled carefully up to the level of dura mater. A 10 μl Hamilton syringe (with 26 G needle) filled with vehicle (citrate buffer) or STZ was inserted through bur hole (4.0 mm deep from the level of dura mater) to reach the lateral ventricle. Vehicle or STZ solution (5 μl) was injected slowly over a period of 20 min. Needle was kept in place for 5 more minutes before withdrawing to avoid backflow of injected solutions. Then, bur holes were sealed using dental acrylic and skin wound was closed with sutures. Antiseptic betadine solution was applied on skin suture and the rats was kept in a warm place for 2 h. Rats received special care during post-surgery period including daily application of betadine antiseptic solution on the wound for 3–4 days and the administration of wet food inside the cage. Rats were housed individually in separate cages until the end of experiment.

### 2.5 Evaluation of cognitive behavior using Morris water maze (MWM)

All rats in 3W and 6W groups belonging to IP and ICV treatments were tested for cognitive performance in the Morris Water maze during 3rd or 6th week post injection, respectively. Cognitive behavior in all rats were assessed between 9:00 a.m. and 4:00 p.m. under optimal room temperature conditions. The assessment was carried out in a quiet, sound-proof room that has a constant temperature of 23 ± 2°C. Cognitive behavior (spatial learning and memory) was evaluated as described in our previous studies (Ishrat et al., 2006, 2009; Ansari et al., 2023) using Morris Water maze (MWM) (Morris, 1984). The MWM consisted of a circular water tank (200 cm diameter and 60 cm height) filled with opaque water (rendered with non-toxic paint) up to 40 cm height and the temperature maintained at 23 ± 2°C. The MWM area was virtually divided into four quadrants (labeled as north, south, east, and west). An escape platform (10 cm diameter) submerged one cm below the water surface in one of the quadrants (platform quadrant). This platform remained fixed on same place for the entire experiment. Before starting the training session, rats were allowed to swim freely in the MWM for 60 sec without the platform. Each rat was then given four learning trials (one from each quadrant) per session. A total nine sessions over a period of 5 days (one session on day 1 and two sessions/day on 2nd–5th day) was given for each rat. Each trial was of 120 sec durations with 60 sec intertrial interval. After reaching the platform, each rat was allowed to remain there for 30 sec before removing from the platform. If a rat failed to reach the platform within 120 sec, then it was gently placed on the platform for the same 60 sec. The time to reach the platform [escape latency (in sec)] and distance travelled (in cm) to reach platform was measured using Ez-video tracking system. Twenty-four hours after the last learning sessions, a probe test (memory retention test) was conducted to test the memory retention. During probe test, platform was removed, and rat was released in the quadrant opposite to platform quadrant and allowed to swim for 30 sec. Time spent in the platform quadrant was recorded.

### 2.6 Isolation of hippocampal synaptosomal fractions

Hippocampal synaptosomal fractions were isolated as previously described (Ansari et al., 2023). Briefly, hippocampal tissues were homogenized in chilled isolation buffer (sucrose 0.25 M, pH 7.2) containing 1.0 mM EDTA, 10 mM HEPES and a cocktail of phosphatase and protease inhibitors (5.0 mg/ml aprotinin, 4.0 mg/ml leupeptin, 4.0 mg/ml pepstatin, 20.0 mg/ml trypsin inhibitor). The homogenates were centrifuged at 1,350 × *g* at 4°C for 5 min and the supernatants were centrifuged at 21,200 × *g* at 4°C for 10 min. The pellets were then resuspended in isolation buffer and loaded onto a sucrose gradient (1.0 M, pH 8.0; and 1.18 M, pH 8.5) and centrifuged at 85,500 × *g* at 4°C for 1 h. Synaptosomal fraction at the interface of sucrose gradient was collected, washed and resuspended in isolation buffer and centrifuged at 32,000 × *g* at 4°C for 20 min. Total protein concentration in isolated synaptosomal fractions was estimated using Pierce^®^ BCA protein assay reagents (Pittsburgh PA, USA).

### 2.7 Estimation of markers of oxidative stress, reduced GSH and TBARS

Glutathione contents and the levels of TBARS in synaptosomal fractions were determined as previously described (Ansari et al., 2008a,b). For the determination of GSH levels, synaptosomal samples were mixed with 4% sulfosalicylic acid in a 1:1 ratio, incubated at 4°C for 1 h and centrifuged at 1,200 × *g* for 15 min. The supernatant was mixed with a reaction mixture containing 0.1 mM 5,5′-dithiobis(2-nitrobenzoic acid) and 0.1 M phosphate buffer (pH 7.4) in a total volume of 250 μl (in a 96-well-plate) and the absorbance was measured at 412 nm. GSH (mg^–1^ of protein) levels in each sample were calculated using a molar extinction coefficient of 13.6 × 10^3^ M^–1^ cm^–1^. For the assessment of TBARS, two sets of samples (0.2 mL) were concurrently incubated at 0°C and 37°C for 1 h. After 1 h incubation, 0.2 mL of 10% trichloroacetic acid (TCA) and 0.4 mL of 0.67% thiobarbituric acid (TBA) were added and samples were centrifuged at 3,500 × *g* for 15 min. The supernatants were then incubated in boiling water for 10 min, cooled to room temperature for 30 min and transferred to a 96-well plate (250 μl/well) and absorbance was measured at 535 nm. TBARS levels were calculated using a molar extinction coefficient of 1.56 × 105 M-1 cm^–^1. The rate of lipid peroxidation was expressed as nmoles of TBARS formed h^–1^ mg^–1^ of protein. Values of GSH and TBARS were then expressed as percentage change relative to vehicle-treated rats (% of control).

### 2.8 Immunoblotting analysis of pre- and post-synaptic proteins

The expression of key synaptic proteins involved in mediating synaptic function and plasticity (synapsin, synaptophysin, GAP-43, SNAP-25, drebrin, SAP-97 and PSD-95) were analyzed by immunoblotting as previously described (Ansari et al., 2008a,b), with some modifications. Synaptosomal fractions were diluted with equal volume of lysis buffer (50 mM Tris–HCL, pH 7.4, 1 mM EDTA, 1% Triton X-100, 10% glycerol including the above-mentioned protease inhibitors) and centrifuged at 13,000 × *g* for 10 min and the supernatant was used for immunoblotting. Samples (20 μg protein) were mixed with equal volume of 1x loading buffer (1:1) and loaded into a gradient gel [(4–20% Tris–HCl), (Bio-Rad)]. Proteins were transferred to a nitrocellulose membrane using a semidry transfer system (Bio-Rad) in transfer buffer (25 mM Tris, 150 mM glycine, and 20% MeOH) for 2 h at 15 V. Thereafter, the blots were stained with ponceau staining to ensure equal transfer of proteins. The blots were then blocked with 5% fat free dry milk in Tris-buffered saline Tween-20 (TBST). Primary antibodies against synapsin, synaptophysin, GAP-43, SNAP-25, drebrin, SAP-97 or PSD-95 and, β-actin as a loading control, were added at 1:1000 dilution and blots were incubated overnight at 4°C. The blots were then incubated with alkaline phosphatase conjugated secondary antibody at 1:8000 for 1 h and developed using BCIP/NBT substrate (Sigma Fast tablets). Finally, the blots were dried, scanned and band intensities were quantified with ImageJ. Protein expression was expressed as a percentage change compared to vehicle group (% of control).

### 2.9 Statistical analysis

Differences between group means were evaluated using one-way analysis of variance (ANOVA) followed by Bonferroni’s multiple comparison test. A two-way ANOVA followed by Bonferroni *post-hoc* test was used for comparisons between treatment methods (IP versus ICV) time post injection (Treatment X Time). The associations between different synaptic proteins and cognitive loss were evaluated using Pearson’s correlation followed by Gaussian distribution (Graph Pad Prism 10, SAS Institute) and expressed in linear regression plots. Alpha was set at 0.05 and data expressed as means ± SD.

## 3 Results

### 3.1 IP-STZ and ICV-STZ administration induces impairments in rat’s cognitive performance

#### 3.1.1 Learning behavior

**FIGURE 1:**
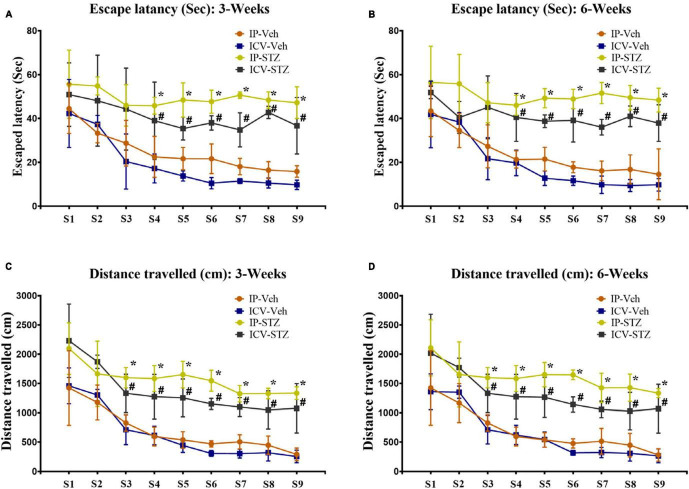
**(A,B)** Escape latency and **(C,D)** distances traveled by rats to reach platform during learning sessions at 3 and 6 weeks after vehicle or STZ (IP or ICV) injection. Rats in IP-STZ and IVC-STZ groups took significantly longer time to reach the platform and traveled a longer distance in 3rd, 4th, and 5th day of learning sessions. ICV-STZ infused rats demonstrated significant learning deficit, indicated by longer escape latency and longer distance traveled, compared to the vehicle treated groups (*p* < 0.05). STZ injected rats showed significant inadequacy in learning during 3rd, 4th, 5th day of learning sessions (IP-Veh-3W and -6W vs. IP-STZ-3W & -6W, *p < 0.05; ICV-Veh-3W & -6W vs. ICV-STZ-3W & -6W, ^#^*p* < 0.05). Data are mean ± SD of *n* = 10 rats/group.

Intraperitoneal- and ICV- STZ injected rats exhibited clear insufficiency in their learning performance indicated by increased latency to escape onto the hidden platform during the water maze learning sessions at 3W and 6W after STZ injection (Figures 1A, B). Latencies to reach the platform gradually decreased in control and STZ treated groups from session 1 to 9 (*p* < 0.001, Two-way repeated measures ANOVA). Mean values of escape latency were, however, significantly prolonged in IP-STZ and ICV-STZ group’s learning sessions on 3rd, 4th, and 5th days (at 3W and 6W post injection) as compared to vehicle controls (*p* < 0.05, Two-way repeated measures ANOVA, Bonferroni’s multiple comparison test). The effects of STZ injection on the distance traveled before the rat escaped on to the platform showed a similar trend to the escape latency (Figures 1C, D). The distance traveled to reach the platform also gradually declined in control and STZ treated groups from the session 1 to 9 (*p* < 0.001, Two-Way repeated measures ANOVA). IP- and ICV-STZ treated rats demonstrated a poor learning performance as indicated the longer distance traveled to reach the platform. The mean distance traveled was significantly protracted in IP-STZ and ICV-STZ groups in learning sessions of 3rd, 4th, and 5th day (at 3W and 6W post STZ injection) compared to vehicle groups (*p* < 0.05, Two-way repeated measures ANOVA, Bonferroni’s multiple comparison test). ICV-injection of STZ was more potent in impairing the learning performance over IP-injection. The escape latency and the distance travelled in vehicle treated rats were not significantly different between ICV-Veh-3W and ICV-Veh-6W. In addition, there was no significant difference between IP-Veh treated or ICV-Veh treated groups at 3W and 6W.

#### 3.1.2 Memory retention

**FIGURE 2:**
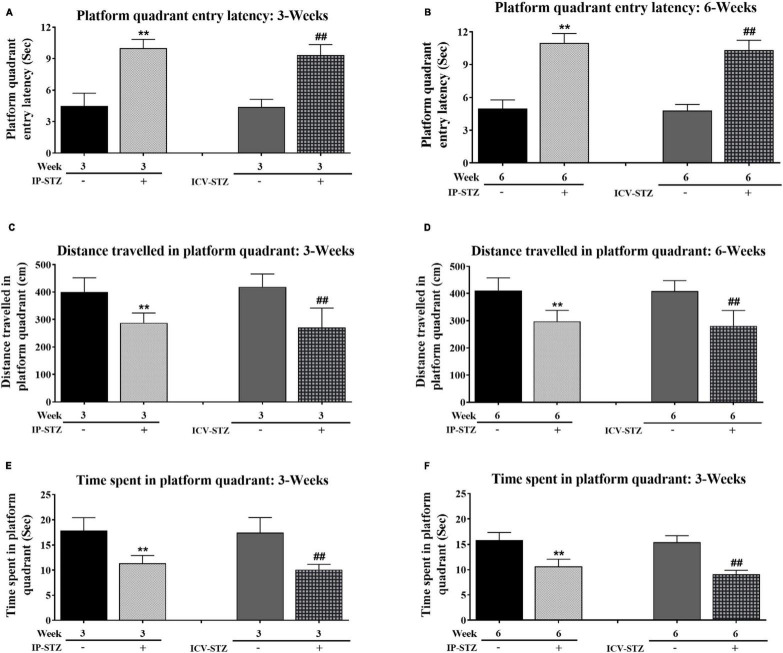
**(A,B)** Platform quadrant entry latency and **(C,D)** distances traveled in platform quadrant, and **(E,F)** time spent in platform quadrant by rats during probe test. Rats in IP-STZ and IVC-STZ groups’ demonstrated significantly longer entry latency into platform quadrant and traveled shorter distance during probe test. STZ infused rats demonstrated significant deficit in memory retention, indicated by longer entry latency, shorter distance traveled, and lesser time spent in in the platform quadrant, compared to the vehicle groups (*p* < 0.01). STZ injected rats showed significant inadequacy in memory retrieval in probe test (IP-Veh-3W & -6W vs. IP-STZ-3W & -6W, **p < 0.01; ICV-Veh-3W & -6W vs. ICV-STZ-3W & -6W, ^##^*p* < 0.01). Data are mean ± SD of *n* = 10 rats/group.

During probe test, IP- and ICV- STZ injected rats exhibited clear insufficiency in their memory performance indicated by increased entry latency to platform quadrant of Morris water maze [3W: *F*(4,36) = 97.071, *p* < 0.001 and 6W: *F*(4,36) = 178.91, *p* < 0.001] (Figures 2A, B). The mean values of platform quadrant entry latency were significantly prolonged in IP-STZ and ICV-STZ groups (at 3W and 6W after STZ injection), during probe test (memory retention test), as compared to vehicle controls (*p* < 0.01). The effects of STZ injection on the distance traveled in platform quadrant showed a dissimilar trend to the entry latency (Figures 2C, D). The distance traveled in the platform quadrant was significantly (*p* < 0.01) declined in STZ treated groups. IP- and ICV-STZ treated rats demonstrated a poor memory retention indicated shorter distance traveled in the platform quadrant. The mean distance traveled was significantly lesser in IP-STZ and ICV-STZ groups (at 3W and 6W post STZ injection) in sessions on 3rd, 4th, and 5th day compared to vehicle groups [3W: *F*(4,36) = 20.724, *p* < 0.001 and 6W: *F*(4,36) = 22.773, *p* < 0.001]. ICV-injection of STZ was more potent in impairing the memory retention over IP-injection. The entry latency and the distance traveled in the platform quadrant were not significantly different between vehicle treated rats of 3W and 6W (IP-Veh treated or ICV-Veh treated groups). IP- and ICV-STZ injected rats did not remember the precise location of the platform during memory retention test. These rats spent less time to explore the quadrant that had the platform in the training session but removed during probe test. Rat’s memory function was significantly impaired in IP-STZ and ICV-STZ groups [3W: *F*(4,36) = 34.241; *p* < 0.001 and 6W: *F*(4,36) = 80.752; *p* < 0.001] (Figures 2E, F).

**FIGURE 3:**
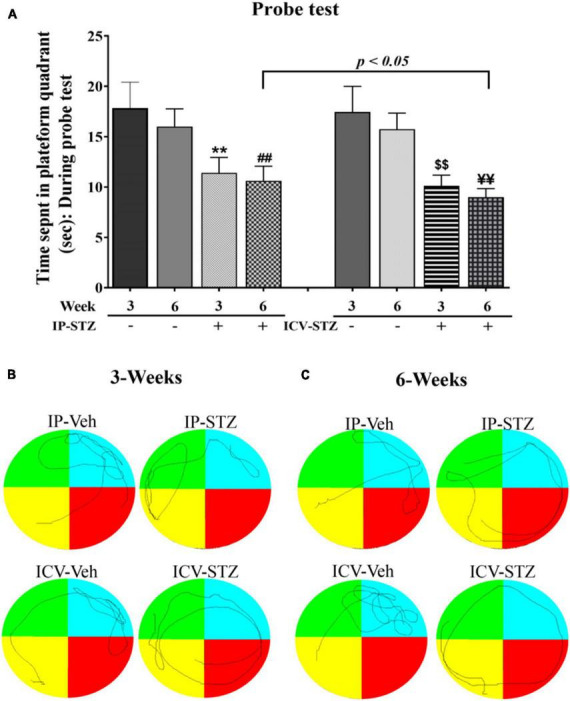
**(A)** STZ infused rats demonstrated significant deficit in memory retention, indicated by longer distance traveled outside the platform quadrant. Time spent by the rats in the platform quadrant during probe test in different groups of vehicles and STZ (IP and ICV) injection. Both the IP-STZ and ICV-STZ injected rats showed significant deficiency in memory retrieval as compared vehicle groups. In addition, ICV-STZ treated rats demonstrated significant deficits in memory retrieval (shorter time spent in the platform quadrant) than IP-STZ treated rats, at 6W (*p* < 0.05). IP-Veh-3W vs. IP-STZ-3W, **p < 0.01; IP-Veh-6W vs. IP-STZ-6W, ^##^*p* < 0.01; ICV-Veh-3W vs. ICV-STZ-3W, ^$$^*p* < 0.01; ICV-Veh-6W vs. ICV-STZ-6W, ^¥¥^*p* < 0.01. Data are mean ± SD of *n* = 10 rats/group. Representative track of one animal from each group during probe test **(B,C)**.

Note that STZ injected rats (IP-STZ and ICV-STZ) at both 3W and 6W less explored the platform quadrant; they explored all four quadrants unlike their respective controls who explored platform quadrant, entering adjacent quadrant once or twice. A comparison of time spent in platform quadrant during memory retention between ICV-STZ and IP-STZ shows significant (*p* < 0.05) higher memory deficiency in ICV-STZ groups as compared to IP-STZ group [*F*(8,72) = 132.04; *p* < 0.001] (Figure 3A) perhaps due to a direct lesion in the cerebral cortex/hippocampus in ICV-STZ group. Finally, there was no significant difference in the mean values of time spent in the platform quadrant during the probe test when compared between vehicle groups, at 3W and 6W groups administered IP or ICV. A representative video tracking of one rat from each group during probe test is showing in the Figures 3B, C.

### 3.2 IP-STZ and ICV-STZ treatment enhances oxidative stress in hippocampus

The levels of GSH and TBRAS were measured in hippocampal synaptosomal fractions isolated from the hippocampi of both IP and ICV STZ groups to assess the extent of oxidative damage. The level of GSH was significantly decreased [(9, 90) = 22.94, *p* < 0.0001] and the levels of TBARS was significantly increased [(9, 90) = 29.27, *p* < 0.0001] in a time dependent manner post-STZ injection (Table 1). These changes were observed as early as 1-week post STZ-injection. GSH levels in IP-STZ-1W group was 82.18% of vehicle control (*p* < 0.05) and in ICV-STZ-1W GSH levels were 72.86% of vehicle control (*p* < 0.01) (Table 1). GSH levels reached a lowest level by 6W (51.36%; *p* < 0.01) in both IP and ICV-STZ treated rats. Moreover, there was no significant difference in GSH levels between the different treatment methods (IP or ICV). TBARS levels were 142.5% (*p* < 0.01) and 177.2% (*p* < 0.01) in IP-STZ-1W and in ICV-STZ-1W, respectively (Table 1) In addition, TBARS levels were significantly different between the different treatment methods. ICV-STZ resulted in a significant [(3, 90) = 12.86, *p* < 0.0001] increase in TBARS levels compared to IP-STZ at 3W (*p* < 0.01) and 6W (*p* < 0.05). Overall, this data suggests failure of internal antioxidant defense system in hippocampal synaptosomes in response to STZ administration IP or ICV. This could possibly be due to imbalance in the levels of antioxidants and oxidants.

**TABLE 1:** Glutathione (GSH) and thiobarbituric acid reactive substance (TBARS) in the hippocampal synaptosomes isolated from the rats following IP or ICV vehicle or STZ injection.

Time after treatment	GSH nmol DTNB conjugate/mg/protein	Changes in % of control	TBARS nmol TBARS/min/mg/protein	Change in % of control
IP-Veh-1W	7.73 ± 1.28	100.0 ± 16.6	7.22 ± 1.43	100.0 ± 19.8
IP-STZ-l W	6.35 ± 1.17*	82.2 ± 15.2*	10.29 ± 2.01	142.5 ± 27.8**
IP-STZ-3W	5.16 ± 0.6**	66.7 ± 7.8**	11.75 ± 1.77	162.8 ± 24.5**
IP-STZ-6W	4.38 ± 0.54**	56.8 ± 7.1**	13.13 ± 2.13	181.9 ± 29.5**
ICV-Veh-l W	7.62 ± 1.15	98.6 ± 14.9	7.94 ± 0.97	110.0 ± 13.5
ICV-Veh-3W	7.43 ± 1.28	96.1 ± 16.6	8.74 ± 1.23	121.0 ± 17.1
ICV-Veh-6W	7.18 ± 0.86	93.0 ± 11.1	9.21 ± 1.49	127.6 ± 20.6
ICV-STZ-l W	5.60 ± 1.01^##^	72.5 ± 13.1^##^	12.79 ± 2.23^##^	177.2 ± 30.9^##^
ICV-STZ-3W	4.62 ± 0.51^$$^	59.8 ± 6.5^$$^	14.32 ± 1.92^$$^	198.3 ± 26.6^$$^
ICV-STZ-6W	3.96 ± 0.59^¥¥^	51.3 ± 7.6^¥¥^	16.21 ± 1.65^¥¥^	224.5 ± 22.9^¥¥^

Levels of GSH and TBARS (Mean ± SD of 10 rats/group; values and changes in% of control) in STZ and vehicle treated (IP or ICV) rats. Note there is time dependent decrease in GSH and a significant increase in TBARS in both the IP-STZ and IVC-STZ groups compared to respective vehicle treated control groups. IP-Veh vs. other groups,

*p < 0.01,

**p < 0.01; ICV-Veh-1W vs. ICV-STZ-1W, ^##^p < 0.01; ICV-Veh-3W vs. ICV-STZ-3W, ^$$^p < 0.01; ICV-Veh-6W vs. ICV-STZ-6W, ^¥¥^p < 0.01. The increase in TBARS were significantly greater in ICV-STZ groups compared to respective IP-STZ groups (3W-p < 0.01, 6W-p < 0.05).

### 3.3 IP and ICV administration of STZ attenuates the expression of hippocampal pre- and post-synaptic proteins in a time dependent manner

**FIGURE 4:**
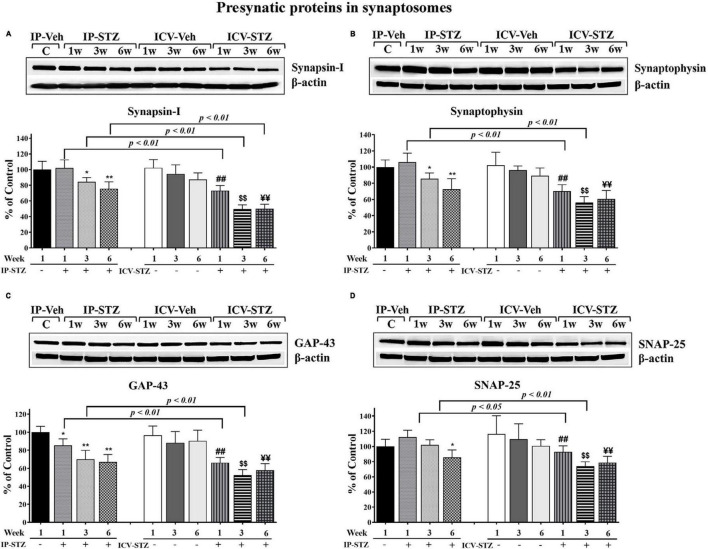
Representative immunoblot of presynaptic proteins, mediators of synaptic plasticity/function synapsin-I, synaptophysin, GAP-43, and SNAP-25 including β-actin, as a loading control, in hippocampal synaptosomes. Quantification of synapsin-I **(A)**, synaptophysin **(B)**, GAP-43 **(C)**, and SNAP-25 **(D)** levels in hippocampal synaptosomal fractions from different groups. The level of presynaptic protein GAP-43 in IP-STZ groups decreased time dependently. The levels of synapsin-I and synaptophysin at 1W, SNAP-25 at 1W and 3W post IP-STZ groups were unaffected compared to IP-vehicle group. Levels of synapsin-I, synaptophysin, GAP-43, and SNAP-25 decreased in a time dependent manner in ICV-STZ groups compared to respective vehicle control groups. The synapsin-I, synaptophysin, GAP-43, and SNAP-25 were significantly lower (*p* < 0.01) in the ICV-STZ groups compared to the IP-STZ groups. IP-Veh vs. other groups, *p < 0.05, **p < 0.01; ICV-Veh vs. ICV-STZ-1W, ^##^*p* < 0.01; ICV-Veh-3W vs. ICV-STZ-3W, ^$$^*p* < 0.01; ICV-Veh-6W vs. ICV-STZ-6W, ^¥¥^*p* < 0.01. Data are mean ± SD of *n* = 10 rats/group.

We evaluated the expression levels of four presynaptic proteins: synapsin-I, synaptophysin, GAP-43, and SNAP-25 following IP and ICV STZ injection (Figure 4). STZ administration (IP and ICV) significantly reduced protein levels of synapsin-I [(9, 90) = 53.22, *p* < 0.0001] (Figure 4A) as compared to the vehicle controls. Moreover, these changes were time dependent: at 1W [IP-STZ, 102.5%, *p* > 0.05; ICV-STZ, 73.2%, *p* < 0.01], at 3W [IP-STZ, 84%, *p* < 0.05; ICV-STZ, 49.5%, *p* < 0.01], and at 6W [IP-STZ, 75.5%, *p* < 0.01; ICV-STZ, 49.95%, *p* < 0.01]. Furthermore, STZ administration via the ICV route was significantly [(3, 90) = 99.65, *p* < 0.0001] more potent in reducing synapsin-I levels as compared to the IP route at all time points post injection (*p* < 0.01). Similarly, the levels of synaptic vesicular protein, synaptophysin, were significantly reduced in STZ treated rats compared to the vehicle controls [(9, 90) = 31.96, *p* < 0.0001] (Figure 4B) and the ICV route of STZ administration was significantly [(3, 90) = 71.44, *p* < 0.0001] more detrimental compared to the IP route, at 1W and 3W (*p* < 0.01). GAP-43 expression was reduced in a time dependent manner following IP and ICV injection of STZ as compared to corresponding vehicle controls [(9, 90) = 35.61, *p* < 0.0001] (Figure 4C). The ICV route of STZ administration exhibited a significantly [(3, 90) = 106.4, *p* < 0.0001] profound effect compared to the IP route at 1W and 3W (*p* < 0.01) [(3, 90) = 106.4, *p* < 0.0001]. Finally, SNAP-25 levels were significantly [(9, 90) = 13.34, *p* < 0.0001] reduced in IP and ICV treated rats. These changes were time dependent in the ICV groups at 1W, 3W and 6W and were significantly [(3, 90) = 22.20, *p* < 0.0001] lower as compared to the IP groups at 1W (*p* < 0.05) and 3W (*p* < 0.01) (Figure 4D).

**FIGURE 5:**
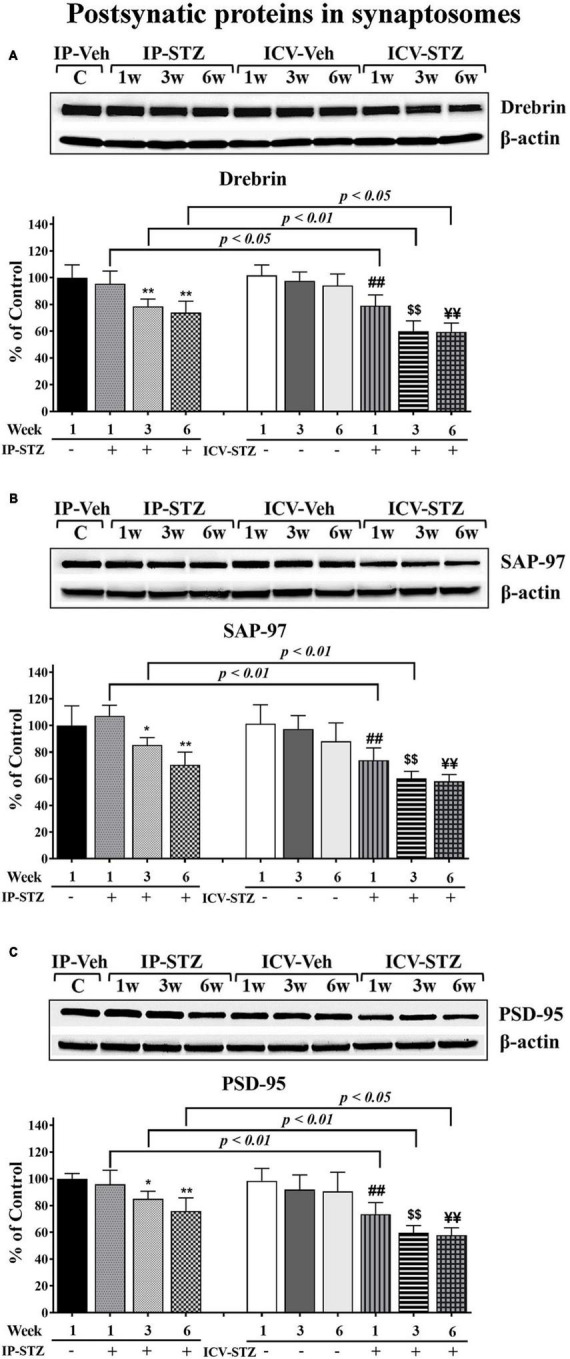
Representative immunoblot of postsynaptic proteins, mediators of synaptic plasticity/function drebrin, SAP-97, and PSD-95 including β-actin, as a loading control, in hippocampal synaptosomes. Quantification of drebrin **(A)**, SAP-97 **(B)**, and PSD-95 **(C)** levels in hippocampal synaptosomal fractions from different groups. The level of postsynaptic proteins drebrin, SAP-97, and PSD-95 were unaffected at 1W post IP-STZ groups compared to IP-vehicle group. Drebrin, SAP-97, and PSD-95 decreased over the time ICV-STZ groups compared to respective vehicle control groups. Levels of these postsynaptic proteins in ICV-STZ groups were decreased similarly at 3W and 6W of time post STZ administration. The drebrin, SAP-97, and PSD-95 were significantly lower in the ICV-STZ groups compared to the IP-STZ groups. IP-Veh vs. other groups, *p < 0.05, **p < 0.01; ICV-Veh vs. ICV-STZ-1W, ^##^*p* < 0.01; ICV-Veh-3W vs. ICV-STZ-3W, ^$$^*p* < 0.01; ICV-Veh-6W vs. ICV-STZ-6W, ^¥¥^*p* < 0.01. Data are mean ± SD of *n* = 10 rats/group.

Furthermore, we evaluated the expression levels of three postsynaptic proteins: drebrin, SAP-97, and PSD-95 following IP and ICV STZ injection (Figure 5). In addition to the observed changes in pre-synaptic proteins, STZ administration IP and ICV reduced the expression of post-synaptic proteins drebrin, SAP-97 and PSD-95. STZ administration significantly reduced drebrin protein expression [(9, 90) = 40.61, *p* < 0.0001] in hippocampal synaptosomes. These changes were time dependent: at 1W [IP-STZ, 95%, *p* > 0.05; ICV-STZ, 79%, *p* < 0.01], at 3W [IP-STZ, 78.56%, *p* < 0.01; ICV-STZ, 60%, *p* < 0.01], and at 6W [IP-STZ, 73.9%, *p* < 0.01; ICV-STZ, 59.5%, *p* < 0.01] (Figure 5A). Moreover, the ICV route demonstrated a significant (*p* < 0.05) detrimental effect over the IP route. Likewise, STZ administration significantly reduced SAP-97 protein expression in hippocampal synaptosomes [(9, 90) = 30.22, *p* < 0.0001]. STZ effects were time dependent and were significantly [(3, 90) = 60.65, *p* < 0.0001] more profound when STZ was administered via the ICV route (*p* < 0.01, at 1W and 3W vs. IP-SZT) (Figure 5B). Finally, STZ administration IP and ICV significantly [(9, 90) = 30.19, *p* < 0.0001] reduced PSD-95 in hippocampal synaptosomes. Time course comparisons [(3, 90) = 22.20, *p* < 0.0001] revealed significant reductions in PSD-95 levels over time that were significantly (*p* < 0.05) lower in the ICV-STZ groups relative to the IP-STZ groups at 1W, 3W, and 6W (Figure 5C).

### 3.4 IP-STZ and ICV-STZ induced oxidative stress and changes in pre- and post-synaptic proteins positively correlated with cognitive impairments

**FIGURE 6:**
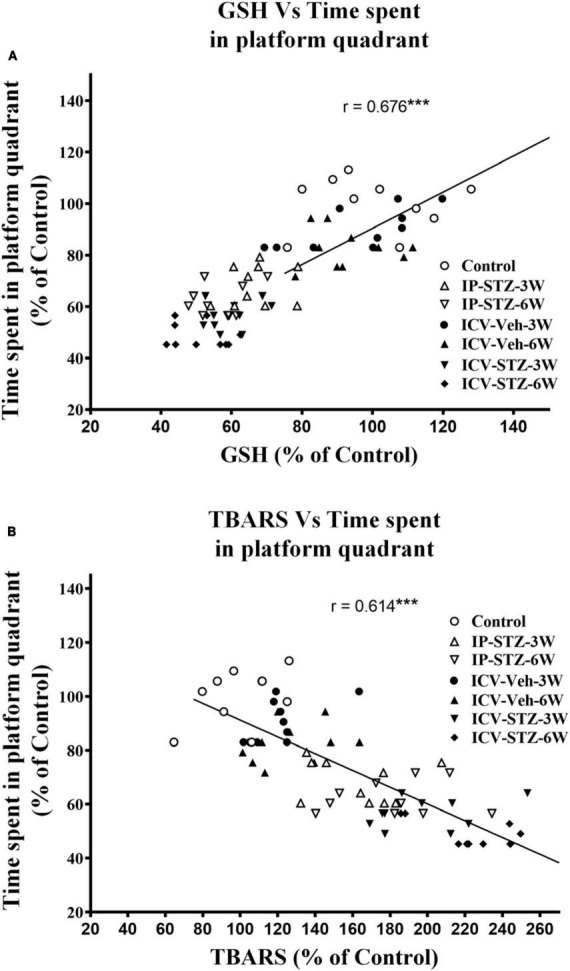
Correlation between GSH **(A)** and TBARS **(B)** levels in response to STZ injections and cognitive performance (time spent in platform quadrant during probe test) in MWM test in different treatment groups. Decreased GSH (antioxidant) level correlated positively with decreased time spent in platform quadrant during probe test (*r* = 0.676), tested at 24 h after last learning session. Increased TBARS (marker of oxidative damages) level correlated negatively with procurement of memory, as searching platform (*r* = 0.614). ***p < 0.0001, *n* = 10 rats/group.

**FIGURE 7:**
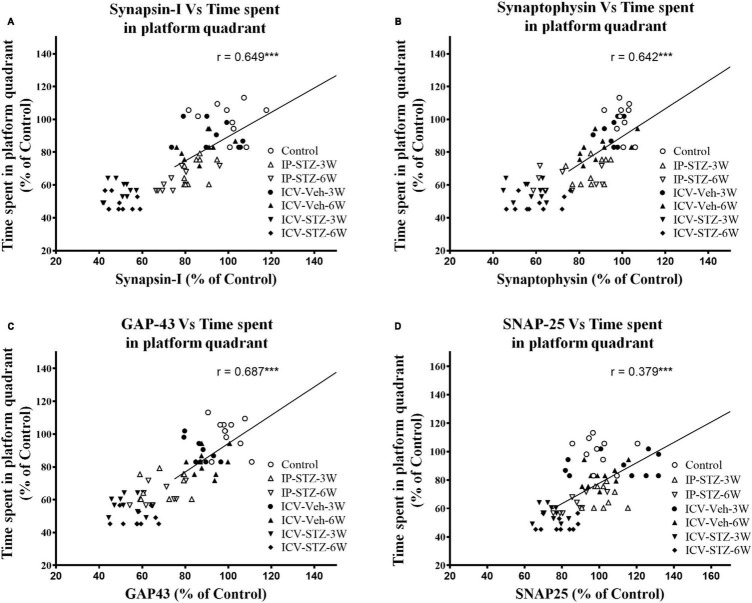
Correlation between changes in presynaptic proteins [declined synapsin-I **(A)**, synaptophysin **(B)**, GAP-43 **(C)**, and SNAP-25 **(D)**] and cognitive performance (time spent in platform quadrant) in MWM. Decreased levels of synapsin-I (*r* = 0.849), synaptophysin (*r* = 0.426), GAP-43 (*r* = 0.687) and SNAP-25 (*r* = 0.379) are correlated positively with decreased spatial memory procurement, tested 24 h after last learning session. As presynaptic proteins are decreased spatial memory acquisition declined. ***p < 0.0001, *n* = 10 rats/group.

**FIGURE 8:**
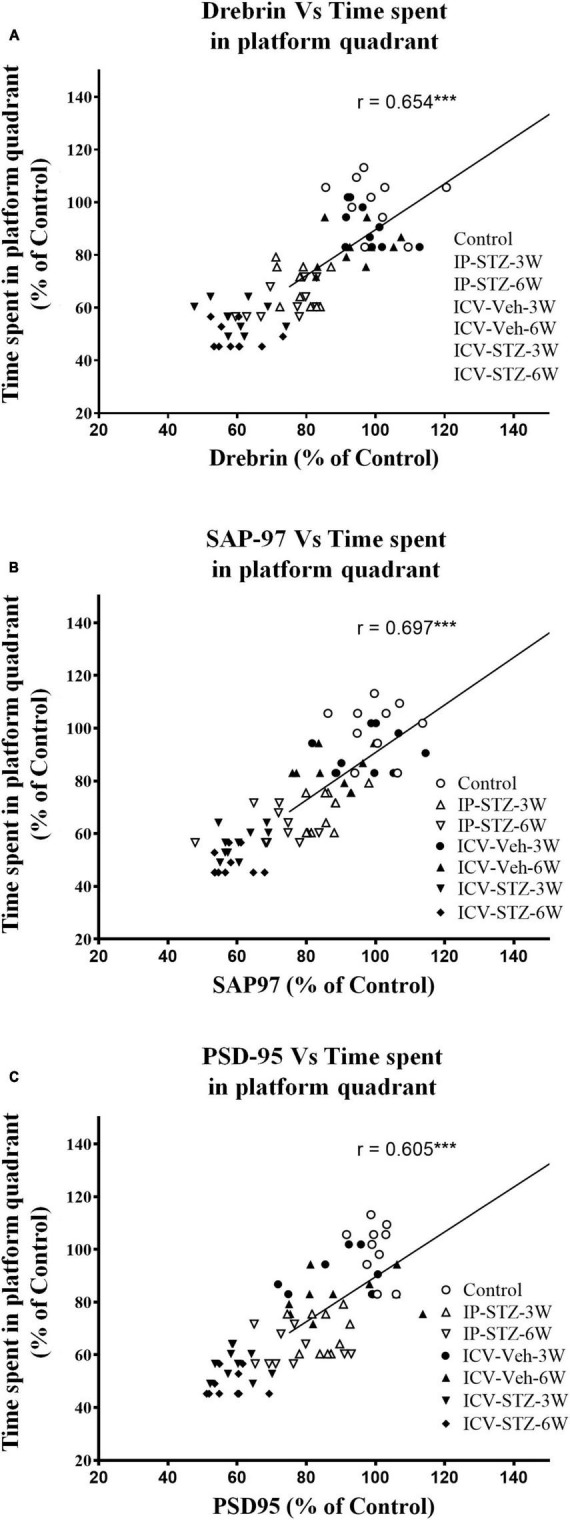
Correlation between changes in postsynaptic proteins [declined drebrin **(A)**, SAP-97 **(B)**, and PSD-95 **(C)**] and cognitive performance (time spent in platform quadrant) in MWM. There is a positive correlation with the level of postsynaptic proteins for the less time spending in the platform quadrant during probe test. Decreased levels of drebrin (*r* = 0.654), SAP-97 (*r* = 0.697), and PSD-95 (*r* = 0.605) are correlated positively with decreased spatial memory procurement in MWM, tested 24 h after last learning session. ***p < 0.0001, *n* = 10 rats/group.

We evaluated the possible relationship between changes in the level of GSH and TBARS, the marker of oxidative stress, and key components in pre- and post-synaptic regions with rats cognitive performance (memory retention in probe test in the MWM). As the level of GSH decreased in hippocampal synaptosomes, the time spent in the platform quadrant significantly decreased (*r* = 0.676; *p* < 0.001, Figure 6A). Moreover, as the level of TBARS increased, time spent in the platform quadrant significantly decreased (*r* = 0.614; *p* < 0.001, Figure 6B). In addition, we evaluated the association between cognitive performance during probe test and the levels of pre- and post-synaptic proteins in hippocampal synaptosomes. There was a significant association (*p* < 0.001) between cognitive impairments and decreased level of synapsin-I (*r* = 0.649; Figure 7A), synaptophysin (*r* = 0.642; Figure 7B), GAP-43 (*r* = 0.687; Figure 7C), SNAP-25 (*r* = 0.379; Figure 7D), drebrin (*r* = 0.654; Figure 8A), SAP-97 (*r* = 0.697; Figure 8B), and PSD-95 (*r* = 0.605; Figure 8C) in hippocampal synaptosomes following STZ injection.

## 4 Discussion

This study evaluated the effect of early changes in oxidative stress and presynaptic (synapsin-I, synaptophysin, GAP-43, and SNAP-25) and postsynaptic (drebrin, SAP-97, and PSD-95) proteins in response to IP and ICV administration of STZ and correlated these changes with rat’s cognitive performance. Our data revealed enhanced oxidative stress and reduced expression of pre- and post-synaptic hippocampal proteins in response to IP and ICV administration of STZ. Our data are consistent with previous data in rodents (Ansari et al., 2023) and in AD patients (Ansari and Scheff, 2010; Scheff et al., 2015, 2016). Nonetheless we demonstrate novel early changes starting at 1W, 3W and 6W post STZ injection, IP or ICV. These findings provide mechanistic insights into the development of dementia and highlight potential therapeutic opportunities.

In addition, this study evaluated the effect of early impairments in insulin signaling pathway on hippocampus dependent cognitive behavior, spatial learning/memory, in IP-STZ and ICV-STZ injected rats. All rats, except in groups of 1W, were evaluated for cognitive performance (learning and memory) using the MWM. The MWM test, a hippocampus dependent task, a widely accepted technique for the evaluation of cognitive behavior and spatial learning/memory in rodents (Morris, 1984). Our data suggested that IP or ICV-STZ administration in rats results in a significant time dependent deterioration of learning and memory and indicated that impaired insulin signaling contributes to the development and progression of dementia as in sAD. ICV administration of STZ, however, exhibited a profound effect over the IP route (Figures 1–3). Our data are consistent with previous studies reporting deteriorated cognition following impaired insulin signaling in rodents 4 weeks post IP-STZ (Zhou et al., 2007; Tian et al., 2016) and 2 weeks post ICV-STZ administration (Rai et al., 2014).

We have previously demonstrated significant synaptic changes in brain tissues from patients with progressive AD (Ansari and Scheff, 2010; Scheff et al., 2015, 2016). Mechanistically, these changes involved loss of synaptic counts, reduced levels of synaptic proteins, and increased oxidative stress in different regions of the AD brains (Ansari and Scheff, 2010; Scheff et al., 2015, 2016). We have recently reported a time dependent increase in oxidative stress markers following IP and ICV-STZ injections (Ansari et al., 2023). Oxidative stress contributes to neurological disorders including AD and associates with ROS formation in neurons and synaptosomes (Cardoso et al., 1998; Butterfield and Boyd-Kimball, 2005; Ansari and Scheff, 2010). Neuronal membranes are rich in polyunsaturated fatty acids and highly susceptible to oxidative damages. This has been implicated in regional neurodegenerative processes in the cerebral cortex of AD (Hensley et al., 1995; Culmsee and Landshamer, 2006). Mitochondria dysfunction is associated to the dysregulation of intracellular Ca^2+^ homeostasis and increased formation of ROS in synaptosomes (Sheehan et al., 1997; Zhu et al., 2004; Ansari et al., 2006; Ansari and Scheff, 2010). Reduced antioxidant GSH and elevated TBARS levels were reported in cerebral tissues of AD models (Balazs and Leon, 1994; Bains and Shaw, 1997; Ansari et al., 2006; Ansari and Scheff, 2010). In the present study, we report a significant increase in oxidative stress (decreased GSH and increased TBARS) in hippocampal synaptosomes from IP-STZ and ICV-STZ treated rats (Table 1). In addition, these changes were exacerbated with time. The occurrence of utmost changes was observed 6 weeks following IP or ICV STZ injection indicating early changes in response to impaired insulin function and its progression over time.

Hyperglycemia results in encephalopathy and exacerbates other neurologic conditions including stroke (Zhang et al., 2010) and AD. Insulin resistance and AD aggravated each other (Park, 2011). Chronic hyperglycemia not only aggravates changes in synaptic morphology and cognitive loss (Zhou et al., 2018), but also may impair cytoprotective mechanisms (Santos et al., 2014). Hyperglycemia modifies synaptic proteins (Garcia-Caceres et al., 2008) and impairs cognition in rodents (Tian et al., 2016). Previous studies demonstrated early (4W) morphological and functional alterations in response to hyperglycemia that aggravated over time. Enhanced synaptosomal lipid peroxidation (Mooradian et al., 1990), reduced AMPA binding to GluR1 (Gagne et al., 1997), attenuated Na^+^/K^+^-ATPase activity (Franzon et al., 2005; Ishrat et al., 2009) were proposed as possible mechanisms. Moreover, the loss of hippocampal LTP attenuated learning/memory in rodents and synergistically interacted with diabetes and dementia (Wang et al., 2015). In addition, reduced expression of insulin receptors (IRs) in synaptic membranes was accompanied by reduced learning/memory (Dou et al., 2005). Interestingly the loss of dendritic spine densities occurs prior to neuronal death (Sanchez-Varo et al., 2012; Ishizuka and Hanamura, 2017) and associates with the loss of synaptic proteins (Kojima and Shirao, 2007; Munsie and Truant, 2012; Shaw and Bamburg, 2017). Our data suggest early, prior to 3W, reduced expression of pre- and post- synaptic proteins in IP- and ICV-STZ treated rats. These findings provide insights into early neurological alterations in response to altered insulin signaling induced by STZ administration.

Streptozotocin infusion in rodents results in the development of sAD phenotypes, including hyperphosphorylation of tau proteins and loss of synaptic plasticity in the cerebral cortex (Rajasekar et al., 2017; Cheng et al., 2019; Gao et al., 2020; Huang et al., 2020; Ide et al., 2020; Latina et al., 2021). The onset of sAD-like pathological changes in response to impaired insulin signaling induced by diabetes is a gradual process that takes a considerable amount of time to develop. However, such alterations can be observed within 2–4 weeks if STZ is administered directly into the brain (Rai et al., 2014; Ahn et al., 2020). sAD phenotype involves increased inflammation, oxidative stress, synaptosomal/mitochondrial dysfunction, and apoptosis in the cerebral cortex (Rai et al., 2014). Two weeks post ICV-STZ infusion, rats demonstrated decreased expression of postsynaptic markers (like, PSD-95), but not presynaptic markers (like, synaptophysin and SNAP-25) increased inflammation and oxidative stress (Rai et al., 2014). Changes in synaptic markers and cognitive behavior, however, were not detected earlier than 2 weeks (at 7–9 days) (Rai et al., 2014). We have previously demonstrated that cognitive loss occurs 3–6 weeks post STZ administration IP or ICV, suggesting early damage to the synaptosomes (Ansari et al., 2023). Henceforth we examined the expression levels of pre- and post-synaptic proteins at 1W, 3W and 6W post STZ injection. Our data clearly suggested attenuated expression of pre- and post-synaptic proteins at early stages of the disease (Figures 4, 5). We observed reduced expression of pre- and post- synaptic proteins by 1W in ICV-STZ model and by 3W in IP-STZ model. However, reduced expression of GAP-43 in IP-STZ was within 1W post injection. Increased oxidative stress in STZ treated rats indicated by decreased GSH and increased TBARS, however, was detected 1W post injection. This implicates oxidative stress as a predisposing factor to reduced expression of pre- and post-synaptic proteins in IP-STZ treated rats. In contrast, however, our results demonstrate increased oxidative stress and reduced expression of synaptic proteins 1-week post ICV-STZ injection. This early increase in oxidative stress is consistent with our previous study in rats (Ansari et al., 2023) and in patients with AD-like pathology (Ansari and Scheff, 2010, 2011). However, impaired memory and loss of synaptic proteins (synaptophysin, and PSD-95) were evident 20 weeks post IP-STZ administration (Wang et al., 2022).

Streptozotocin administration downregulated IRs expression and signaling in the cerebral cortex starting at 3W (Wang et al., 2018; Baltaci et al., 2022). Progressive loss in synaptic function (Shonesy et al., 2012; Gao et al., 2014; Lu et al., 2017), hippocampal LTP (Hashemi-Firouzi et al., 2017) and cognitive behavior (Qi et al., 2021) were detected over time. STZ-mediated neuronal loss in the hippocampus was suggested as a possible mechanism of reduced presynaptic proteins and hippocampal volume (Zappa Villar et al., 2020). Our findings indicate that a substantial decrease in synaptic proteins during the early stages of the disease may contribute to neuronal loss and subsequent reduction in tissue volume.

Impaired insulin function in the brain results in sAD-like phenotypes (Ahn et al., 2020), facilitates loss of synaptic plasticity and causes cognitive deficits (Kim et al., 2013). Insulin signaling mediates Ca^2+^ dependent calmodulin pathways that regulate LTP in tissues (Coleman and Biederer, 2018; Stein et al., 2020). Calmodulin-like Ca^2+^- binding proteins are highly expressed in the brain and interact with several synaptic proteins that involved in the cytoskeletal organization (Kim et al., 2017; Coleman and Biederer, 2018). Presynaptic proteins (synapsin-I, synaptophysin, GAP-43, and SNAP-25) mediate the transfer, docking of vesicles, and release of neurotransmitters in the presynaptic region (Tarsa and Goda, 2002; Zhou et al., 2007; Rai et al., 2014; Wang and Zhao, 2016; Chen et al., 2022). The level of these proteins is strongly correlated with synaptic plasticity (Tarsa and Goda, 2002; Zhou et al., 2007) and cognitive abilities in humans (Hou et al., 2012). During LTP and LTD, dendritic spines undergo dynamic changes based on the type of stimulus received from presynaptic terminals in the hippocampus. Such activity dependent remodeling of synaptic structure and response is thought to be a primary mechanism of cognitive behavior (Holtmaat and Svoboda, 2009; Kim et al., 2017). Moreover, postsynaptic scaffolding proteins mediate anchoring, trafficking, clustering, and diffusion of the receptors into synaptic membrane, which crosstalk with insulin signaling. For example, GSK-3β mediated phosphorylation of PSD-95 is essential for the interaction and internalization of AMPARs into synaptic membranes (Kim et al., 2017) which itself is essential for synaptic plasticity and induction of LTP (Zhao et al., 2013). Postsynaptic protein, drebrin, binding to actin is fundamental to cell dynamics in postsynaptic regions. Displacement or lack of drebrin at its actin-biding site (Minamide et al., 2000; Zhao et al., 2006) and postsynaptic membranes (Ma et al., 2008) are markedly associated with cognitive impairments in AD (in clinical and preclinical samples) (Zhao et al., 2006; Arsenault et al., 2013). We demonstrate that cognitive loss following impaired insulin signaling is significantly associated to increased oxidative stress and attenuation of pre- and post-synaptic proteins in hippocampal synaptosomes (Figures 6–8). Our data are consistent with previous research demonstrating reduced expression of synaptic proteins 4 weeks following ICV-STZ (Hou et al., 2012) or IP-STZ injection (Zhou et al., 2007; Wang and Zhao, 2016; Chen et al., 2022).

It is important to note that the assessment of synaptic proteins and oxidative stress, used animal models of impaired insulin signaling, which should be taken into consideration when generalizing correlation with synaptic changes and cognitive results. The assessment of synaptic proteins and oxidative stress carried out in the hippocampal synaptosomes were not limited to the hippocampus and its dependent spatial memory. Further studies are required to using similar animal models for the investigation of other factors (like, neurotrophic factors and inflammation, etc.), other cognitive behavior (like, working memory, novel object or place recognition, etc.) and applying different techniques (like, immunohistochemistry, OMICS, and electrophysiology, etc.) to fully corroborate the current observations. Despite these forewarnings, evidence is accumulating that impaired insulin signaling mediated synaptic dysfunction plays a key role in the development of sAD.

## 5 Conclusion

**FIGURE 9:**
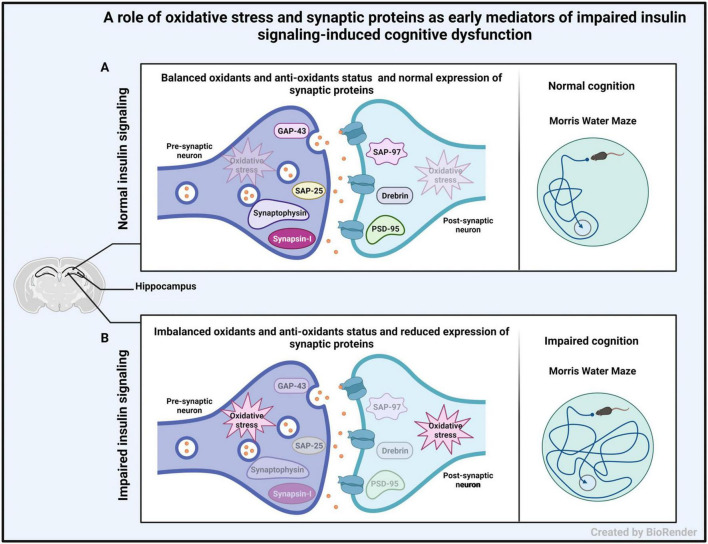
Schematic diagram showing the role of oxidative stress and synaptic proteins as early mediators of altered insulin signaling inducing cognitive dysfunction. **(A)** Representation of hippocampal synapse in control animals having good cognitive function with normal level of oxidative stress and normal expression of pre- and post-synaptic proteins in the hippocampus. **(B)** Representation of hippocampal synapse in animals with impaired insulin signaling (induced by STZ injection), with increased level of oxidative stress and decreased expression of pre- and post-synaptic proteins in the hippocampus. This may lead to cognitive impairment.

We demonstrate enhanced oxidative stress and reduced expression of pre- and post-synaptic proteins in hippocampal synaptosomes from IP and ICV-STZ treated rats. The observed changes were evident early after STZ administration and were more profound upon ICV administration of STZ and correlated with impaired cognition observed in STZ treated rats. Figure 9 illustrates the role of oxidative stress and synaptic proteins as early mediators of altered insulin signaling inducing cognitive dysfunction. As shown in the figure, hippocampal synapse in control animals had balanced oxidants and antioxidants (no oxidative stress) and normal expression of synaptic proteins in the hippocampus with good learning and memory. On the other hand, in animals with impaired insulin signaling, had imbalanced oxidants and antioxidants (oxidative stress) and reduced expression of synaptic proteins in the hippocampus, which might have led to cognitive impairment in them. Thus, our study provides mechanistic insights into the role of oxidative stress and synaptic protein expression as an early mechanism of impaired insulin signaling induced cognitive dysfunction.

## Data availability statement

The original contributions presented in this study are included in this article/supplementary material, further inquiries can be directed to the corresponding author.

## Ethics statement

The animal study was approved by the Animal Ethical Committee, Health Sciences Center, Kuwait University, Kuwait. The study was conducted in accordance with the local legislation and institutional requirements.

## Author contributions

MA: Conceptualization, Data curation, Formal analysis, Investigation, Methodology, Writing—original draft, Writing—review and editing. MR: Data curation, Formal analysis, Investigation, Methodology, Writing—review and editing. AA-J: Formal analysis, Investigation, Methodology, Writing—review and editing.
